# Häusliche Isolation und Quarantäne gut überstehen

**DOI:** 10.1007/s00278-020-00424-y

**Published:** 2020-04-30

**Authors:** Frank Jacobi

**Affiliations:** Klinische Psychologie und Psychotherapie, Psychologische Hochschule Berlin, Am Köllnischen Park 2, 10179 Berlin, Deutschland

**Keywords:** Coronavirusinfektionen, Infektionskontrolle, Psychologischer Stress, Gewaltprävention, Soziales Verhalten, Coronavirus infections, Infection control, Psychological stress, Prevention of violence, Social behavior

## Abstract

Das neuartige Coronavirus SARS-CoV‑2 und die dadurch verursachte Krankheit mit offiziellem Namen „coronavirus disease 2019“ (COVID-19) zwingen weltweit zu drastischen Maßnahmen, die die massenhafte Ausbreitung eindämmen sollen. Hierzu zählt die weitestmögliche häusliche Isolation der Bevölkerung. Häusliche Isolation und Quarantäne sind aber Ausnahmesituationen, die die meisten Menschen noch nicht erlebt haben. Diese gesetzten Maßnahmen können auf die Psyche einwirken und für Betroffene sehr belastend sein. Hier helfen klare, in der Psychologie wissenschaftlich erforschte und bewährte Verhaltensmaßnahmen sowie mentale Strategien, diese Ausnahmesituation zu meistern.

## Abstract (en)

The novel coronavirus SARS-CoV‑2 and the disease caused by it with the official name coronavirus disease 2019 (COVID-19) is forcing the implementation of drastic measures worldwide, which are aimed at containing the massive spread. The measures include domestic isolation of the population as far as possible. Domestic isolation and quarantine are, however, exceptional situations, which most people have not yet experienced. These set measures can have an effect on the psyche and be very debilitating for those affected. Clear behavioral measures and mental strategies, which have been scientifically researched and established in psychology, help to overcome this exceptional situation.

Die COVID-19-Pandemie veranlasst weltweit zu drastischen Maßnahmen. Quarantäne und Abstandhalten der Menschen voneinander sollen – insbesondere schwache, ältere oder körperlich angegriffene – Mitmenschen vor der Ansteckung mit dem Coronavirus schützen. Die Einstellung und das Verhalten aller haben einen wesentlichen Einfluss darauf, wie wir als Gesellschaft diese zuvor nichtdagewesene Herausforderung bewältigen werden.

## Psychologische Hilfen in herausfordernden Zeiten

Das neuartige Coronavirus *SARS-CoV‑2* und die dadurch verursachte Krankheit mit offiziellem Namen „coronavirus disease 2019“ (COVID-19) veranlassen weltweit drastische Maßnahmen, die die rasante massenhafte Ausbreitung eindämmen sollen. Wenn keine bevölkerungsweite Verhaltensänderung eintritt, „… besteht die Gefahr, dass wir in 2 bis 3 Monaten bis zu 10 Mio. Infizierte haben, mit entsprechender Überlastung des Gesundheitssystems“^1^. Dieser Prozess lässt sich allerdings bremsen. Neben den bekannten Hygieneregeln spielt insbesondere das Einhalten zwischenmenschlicher Distanz eine entscheidende Rolle.

Häusliche Isolation und Quarantäne sind Ausnahmesituationen, die die meisten Menschen noch nicht erlebt haben. Diese gesetzten Maßnahmen können auf die Psyche einwirken und für Betroffene sehr belastend sein. Die wichtigsten Stressoren sind – besonders, wenn die Quarantänemaßnahmen länger andauern – Ansteckungsängste, Frustration und Langeweile, Einschränkung der materiellen Versorgung, Fehlinformation, finanzielle Nöte und Stigmatisierung von Betroffenen (Brooks et al. 2020).

Es gibt allerdings klare, in der Psychologie wissenschaftlich erforschte und bewährte Verhaltensmaßnahmen und mentale Strategien, die es ermöglichen, diese Ausnahmesituation zu meistern. Grundsätzlich gilt: Jeder Mensch ist anders und sollte die individuell zur Person und zur Lage passenden Strategien suchen, ausprobieren und umsetzen. In außergewöhnlichen Zeiten kann es zu neuen Belastungen und ungewohnten Emotionen kommen. Es braucht Zeit, sich an diese neuen Umstände und Herausforderungen – einschließlich der Langeweile – zu gewöhnen.

Die folgenden Checklisten enthalten für Sie möglicherweise nicht wirklich neue Erkenntnisse, können aber dennoch Unterstützung bieten, „kühlen Kopf zu bewahren“ sowie für Sie selbst und Ihre Nächsten die besten Strategien zu entwickeln, mit der neuartigen Situation umzugehen. So können Sie Ihren eigenen Beitrag dazu leisten, dass wir die anstehenden Herausforderungen persönlich und als Gesellschaft gemeinsam und solidarisch bewältigen.

## Allgemeine hilfreiche Maßnahmen

**→ Denken wir stets daran, dass wir einen Dienst an der Gemeinschaft tun, wenn wir uns an die Quarantäneempfehlungen und die Richtlinien zur Reduzierung direkter sozialer Kontakte halten!**

Quarantäne und Abstandhalten helfen anderen – insbesondere schwachen, älteren oder körperlich angegriffenen Mitmenschen. Diese Maßnahmen sind in großem Maße sinnvoll, und zwar nicht nur für die anderen: Diejenigen, die etwas für die Gemeinschaft tun, haben weniger Angst. Auch Großzügigkeit gegenüber anderen, denen es aktuell schlechter gehen mag als einem selbst, kann die eigene Gefühlslage verbessern und stabilisieren.

Allein die Sinngebung ist bereits wichtig. Wir zeigen große kollektive soziale Verantwortung durch dieses altruistische Handeln. Wenn uns dies klar ist, fällt es uns leichter, auch mit eigenen Spannungen akzeptierend und respektvoll umzugehen.

**→ Halten Sie eine Tagesstruktur ein!**

Struktur hilft gegen Chaos, gibt Sicherheit und stärkt in Stresssituationen. Vorhersehbarkeit und Selbstwirksamkeit werden dadurch gefördert, und Zustände von Hilflosigkeit werden reduziert. Das bedeutet konkret: nicht im Pyjama bleiben, sondern wie immer aufstehen, sich anziehen, übliche Essens‑, Schlafens‑, Arbeits- oder Lernzeiten (und auch Feierabendzeiten) einhalten. Passen Sie Ihre Tagesstruktur an die aktuelle Situation an.

Setzen Sie sich realistische Ziele und planen Sie Ihren Tag möglichst genau und verbindlich. Aufgabenverwaltungstools können hilfreich sein, insbesondere wenn man nun arbeitsbezogen mehr auf sich allein gestellt ist. Erstellen Sie nicht nur „To-do“-Listen, sondern auch „Done“-Listen und erzählen Sie anderen von Ihrer Zielerreichung.

Durch geplantes Handeln hat man das Gefühl, einer Situation nicht hilflos ausgeliefert zu sein, sondern diese aktiv zu gestalten. Sie könnten z. B. „Projekte“ starten, die Sie bisher aufgeschoben haben – vielleicht sind das auch „kleine Dinge“ wie Tagebuch schreiben, neue Fertigkeiten lernen, aufräumen oder Arbeiten erledigen, die sonst immer liegen geblieben sind. Planen Sie ein Highlight pro Tag, auf das Sie sich freuen können.

Sollte es Ihnen nicht immer gelingen, Ihre Tagesstruktur einzuhalten, ist das allerdings verständlich. Vielleicht ist es Ihnen ja möglich, diesbezüglich nachsichtig mit sich umzugehen.

**→ Konsumieren Sie Medien bewusst und gezielt!**

Wir wissen aus Erfahrung von Menschen, die Katastrophen und Tragödien erlebt haben, dass ein Übermaß an Beschäftigung mit diesen Geschehnissen in den Nachrichten und Meldungen aller Art negative emotionale Folgen haben und die negativen Effekte noch weiterverstärken kann.

Zwar helfen Fakten gegen überschwemmende Gefühle von Kontrollverlust und Hilflosigkeit, denn seriöse und klare Informationen geben Orientierung und Sicherheit. Jedoch kann ununterbrochener Medienkonsum überfordern, indem immer wieder bestimmte Bilder und Schilderungen wiederholt werden. Zudem sind einige Falschinformationen im Umlauf. Deshalb ist es wichtig, den Bezug zur Realität nicht zu verlieren. Im Grunde reicht ein tägliches Update aus einer verlässlichen Quelle aus, um angemessen auf dem Laufenden zu bleiben.

**→ Pflegen Sie Ihre sozialen Kontakte über die Distanz!**

Verbundenheit mit der Familie oder dem Freundeskreis gibt Halt. Auch regelmäßige Treffen mit Kolleginnen und Kollegen per Chat oder Videokonferenz können nett und motivierend sein. Bleiben Sie also in Verbindung, auch wenn Sie sich nicht von Angesicht zu Angesicht nahe sein sollen. Nutzen Sie dazu Telefon, Videochats, soziale Netzwerke & Co. Und scheuen Sie sich nicht, Unterstützung zu suchen, wenn Sie welche benötigen – die allermeisten Mitmenschen helfen gern.

Halten Sie sich bei Ihren sozialen Kontakten von Panikmachern fern. Wenn Sie das Gefühl haben, dass bestimmte Kontakte immer wieder COVID-19-bezogene Informationen wiederholen und dies Ihnen ein ungutes Gefühl gibt, setzen Sie Grenzen und verzichten Sie auch darauf, die nichtpersönlichen, massenweise kursierenden SMS, E‑Mails, Videos, WhatsApp-Nachrichten und Meldungen auf sozialen Medien zur Pandemie zu lesen. Vielleicht ist es Ihnen stattdessen möglich, sich auf gemeinsame positive Inhalte zu fokussieren, etwa „Was hat dich heute gefreut?“ oder „Wofür bist du dankbar?“.

Und: Denken Sie auch an entferntere Verwandte, Nachbarn und Bekannte, die möglicherweise kein soziales Netzwerk haben, und kontaktieren Sie diese, damit sie sich nicht vergessen fühlen und ggf. Hilfebedarf äußern können.

**→ Besinnen Sie sich auf Ihre Stärken!**

Ressourcen helfen, Krisensituationen durchzustehen. Innere Ressourcen sind alles, was Sie an positiven Erfahrungen in Ihrem Leben gemacht haben, alle Probleme, die Sie schon überwunden und gelöst haben, Ihre Stärken und Talente, Ziele und Werte. Ressourcen sind Kraftquellen. Aktivieren und nutzen Sie diese – und haben Sie dabei auch neue Freiräume, Spielräume, Ruhe und Entschleunigung in den eigenen vier Wänden im Blick, die einen wohltuenden Nebeneffekt der zwangsweisen Auszeit, denen viele von uns ausgesetzt sind, darstellen. Das kann beispielsweise sein, sich dem Hobby zu widmen, kreativ tätig zu sein.

Falls Sie schon mal Phasen mit schweren psychischen Problemen hatten, besinnen Sie sich darauf, wie Sie diese begrenzt oder überwunden haben, oder was Sie sich möglicherweise in einer vergangenen oder laufenden Psychotherapie erarbeitet haben.

Auch hier gilt: Sie müssen das nicht in der Zeit, die Sie zu Hause verbleiben, perfektionieren. An manchen Tage wird es Ihnen besser gelingen, Ihre Ressourcen zu nutzen, an anderen Tagen finden Sie vielleicht nur schwer Zugang. Auch das ist kein Grund zu Besorgnis und bedeutet nicht, dass Sie mit der Situation nicht gut umgehen können.

**→ Geben Sie Ihren Gefühlen Raum!**

Wir alle haben unterschiedlichste Gefühle in dieser ungewohnten Situation, z. B. Verwirrung, Angst oder Stresserleben. Diese Gefühle sind absolut verständlich. Beachten Sie diese und versuchen Sie, Ihre Gefühle zu akzeptieren: Unfreiwillig in häuslicher Quarantäne zu sein, kann verschiedene emotionale Reaktionen hervorrufen. Es kann helfen, in solch gefühlsbestimmten Zeiten keine gravierenden Entscheidungen zu treffen, etwa über einen neuen Arbeitsplatz oder die Beendigung einer Beziehung.

Nehmen Sie sich Zeit, um wahrzunehmen und auszudrücken, was Sie fühlen. Manche Menschen schreiben ihre Gefühle gern nieder oder werden kreativ (z. B. malen, musizieren oder meditieren). Häufig hilft es schon, die Situation aus einer anderen Perspektive zu betrachten – wie würde eine nahestehende Person damit umgehen?

Sprechen Sie über Ihre Gefühle, z. B. mit einer hilfreichen Bezugsperson. Sollte diese im näheren Umfeld nicht vorhanden sein, holen Sie sich professionelle Hilfe, z. B. bei Krisendienst, Notfallseelsorge sowie lokalen und überregionalen Hotlines, die derzeit eingerichtet werden. Vermeiden Sie ungesunde Strategien der Gefühlsregulation – z. B. schädlichen Gebrauch von Alkohol und Drogen^2^.

**→ Begrenzen Sie das Grübeln!**

Über etwas intensiv nachzudenken, ist eine von vielen Strategien im Umgang mit Stresssituationen. Ein Zuviel an Grübeln ist jedoch oft kontraproduktiv, da es zusätzlichen Stress verursachen kann. Überlegen Sie sich daher schon im Vorhinein Tätigkeiten, die Sie ausführen können, sollten Sie ins Grübeln verfallen. Machen Sie etwas ganz anderes, das Ihnen guttut. Manche Menschen backen, lesen oder schreiben beispielsweise gern. Auch Humor ist in ernsten und sorgenvollen Zeiten nicht nur erlaubt, sondern geradezu erwünscht.

Übrigens ist es eine hilfreiche Strategie, das Grübeln zu verschieben und auf selbst festgelegte Grübelzeiten zu begrenzen. Nehmen Sie sich, vorab festgelegt, am Tag 10–20 min Zeit, in der Sie so viel grübeln können, wie Sie möchten. Wenn außerhalb dieser Zeiten Sorgen auftauchen, sagen Sie zu sich selbst: „Darüber denke ich nachher (oder morgen) in meiner Grübelzeit nach – aber nicht jetzt.“ Wenn man das regelmäßig übt, dann automatisiert man diesen gedanklichen Prozess und kann sich große Entlastung schaffen. Solche Grübelzeiten sollten übrigens nicht auf den Abend oder die Nacht gelegt werden.

### → Führen Sie einfache Entspannungs- und Achtsamkeitsübungen durch!

Angst und Entspannung können nicht gleichzeitig bestehen. Daher machen Sie Entspannungsübungen; diese reduzieren Ängste und können helfen, achtsam und akzeptierend mit der einengenden Situation umzugehen. Auch im Internet finden Sie Anleitungen für Entspannungsübungen.

*Beispiel*: Eine einfache Übung ist zu bemerken, wo man gerade sitzt und den Kontakt zur Sitzfläche wahrzunehmen. Vielleicht gelingt es Ihnen, kurz die Augen zu schließen und zu spüren, ob Sie im Körper Spannungen wahrnehmen. Stellen Sie sich vor, es verläuft ein Faden entlang Ihrer Wirbelsäule, der Sie aufrichtet. Nun können Sie sich auf Ihren Atem konzentrieren: Wie langsam oder schnell ist dieser? Wo spüren Sie ihn am stärksten – in der Bauchregion, beim Einfließen der Luft in die Nase? Versuchen Sie, einem Atemzug von Beginn des Einatmens bis zum Ausatmen zu folgen. Es geht nicht darum, den Atem in irgendeiner Form zu verändern – er ist so, wie er ist, absolut in Ordnung. Es geht nur darum, ihn wahrzunehmen. Sollten Gedanken auftauchen, versuchen Sie, Ihre Aufmerksamkeit wieder auf den Atem zu lenken. Sie können mit einer Minute beginnen und diese Übung, so oft Sie mögen, wiederholen.

### → Bewegen Sie sich!

Bleiben Sie nicht nur mental, sondern auch körperlich aktiv. Bewegung bewirkt Wunder im Kopf und wirkt sich, wissenschaftlich nachgewiesen, positiv auf unsere Psyche aus. Sport ist auch auf engem Raum möglich: Videos im Internet liefern Anregungen und Trainingsprogramme. Viele Anbieter von Trainingsstudios machen ihre Angebote nun auch online verfügbar. Auch hier gilt: Sicherlich ist es nicht einfach, die gewohnte Routine in den nun veränderten Alltag zu integrieren. Möglicherweise müssen Sie etwas daran ändern, wie Sie gewohnt sind, Sport zu treiben. Trotzdem ändert dies nichts an den positiven Effekten von Bewegung auf Ihre körperliche und mentale Gesundheit.

### → Bei alledem: Denken Sie daran, dass die Situation vorübergehen wird!

Es ist wichtig zu verstehen, dass die aktuelle Pandemie unweigerlich irgendwann vorübergehen wird – auch wenn wir uns darauf einstellen müssen, dass dies nicht in einigen Wochen der Fall sein wird (der Höhepunkt der Probleme wird noch Monate auf sich warten lassen, und wir müssen auch mit mehreren Infektionswellen rechnen). Auf die derzeitigen Gefahren, Ängste und Sicherheitsmaßnahmen werden dennoch mit Sicherheit auch wieder Entwarnung, Neubesinnung und Normalisierung folgen. Es ist nicht damit zu rechnen, dass die Welt wegen COVID-19 oder der daraus erwachsenden Probleme untergeht.

Manches hilft, diesen Prozess der Normalisierung zu beschleunigen – hierzu zählen „… schonungslose Transparenz, besonnene und entschlossene Entscheidungskonsequenz, diszipliniertes Durchhaltevermögen aller, Offenheit für neue Perspektiven wie die Wiederentdeckung solidarischer Werte und das Bewusstsein, in einer globalen Schicksalsgemeinschaft gemeinsam bestehen zu können“ (Ziems 2020).

Unterschätzen Sie nicht die einfachen und bekannten effektiven Möglichkeiten, um Ihr Erkrankungsrisiko jetzt zu vermindern und die Infektion zu verlangsamen (z. B. durch regelmäßiges Händewaschen und Vermeiden von engem zwischenmenschlichem Kontakt). Planen Sie aber auch Aktivitäten, die Sie nach dem Überstehen der jetzigen Situation ausführen möchten.

## Besondere Maßnahmen im Umgang mit Kindern und Jugendlichen

Isolation ist eine Belastung. Das oberste Ziel in der Isolation ist, diese Zeit möglichst stressfrei zu bewältigen (nicht nur für die Kinder, sondern auch für die Erwachsenen). Die Isolation ist nicht dazu da, die Familie besser zu machen. Die Erziehung der Kinder oder die Konfliktbewältigung mit dem Partner sollen in dieser Zeit nicht im Fokus stehen.

- Halten Sie die **gewohnte Tagesstruktur** ein.
- Planen Sie **klare Lern- und Freizeiten**. Bieten Sie Ihrem Kind die Möglichkeit, sich auch mental zu betätigen, z. B. durch Lesen, Schreiben oder Knobelaufgaben.
- Definieren Sie klar **abgegrenzte Stunden, in denen sich jede Person allein beschäftigt**.
- Führen Sie **gemeinsame Aktivitäten** aus.
- Bieten Sie **Rückzugsmöglichkeiten**, um Konflikte zu verhindern bzw. zu reduzieren.
- Auch wenn es keinen adäquaten Ersatz für den Spielplatz oder das Spielen im Freien gibt: Sorgen Sie bei Ihrem Kind im Rahmen der aktuellen Möglichkeiten für **körperliche Betätigung**.
- Eine **gesunde Ernährung** ist immer wichtig, gerade jetzt.
- Erarbeiten Sie **gemeinsame Regeln**, wie die gewonnene Zeit bestmöglich genützt werden kann.
- **Limitieren Sie gemeinsam mit dem Kind die „Screen-Zeiten“** für Fernsehen, Mobiltelefon oder Computer.
- Erklären Sie Ihrem Kind **in altersgerechten Worten** die aktuelle Situation. Wenn Ihr Kind Fragen stellt, beantworten Sie diese ehrlich. Sagen Sie offen, wenn Sie etwas selbst nicht wissen. Sie können dann gemeinsam überlegen, wer Ihnen die gewünschte Antwort geben kann.
- **Kinder haben ein anderes Zeiterleben als Erwachsene.** Malen Sie z. B. einen Kalender und streichen Sie – ähnlich einem Adventskalender – jeden Tag der Quarantäne ab, sodass die Zeitspanne für Ihr Kind greifbarer wird.
- **Akzeptieren Sie, wenn Ihr Kind anhänglicher ist als sonst** und kommen Sie diesem Bedürfnis Ihres Kindes nach. Es braucht gerade jetzt Sicherheit und Geborgenheit.
- **Verzichten Sie darauf, gerade jetzt große Erziehungsmaßnahmen zu setzen und sehen Sie möglichst von Strafen ab**. Versuchen Sie, ihr Kind durch Lob positiv zu verstärken und zu erwünschtem Verhalten zu motivieren.
- **Bewahren Sie sich eine positive Grundhaltung**: Diese kann sich auch auf Ihr Kind übertragen und vermittelt Zuversicht und Sicherheit.

## Maßnahmen gegen das Auftreten von Konflikten

In engen räumlichen Verhältnissen entsteht „Dichtestress“. „Unter *Lagerkoller* einzelner oder mehrerer Personen versteht man umgangssprachlich einen vorübergehenden psychischen Erregungszustand bei zwangsweiser Lagerunterbringung, wie er v. a. in Gefängnissen, Kasernen, Kriegsgefangenenlagern, Deportierungslagern, Flüchtlingslagern, Notunterkünften und Katastrophenschutzlagern bei anhaltend belastenden und unabsehbar lange andauernden Bedingungen vorkommt. ‚Koller‘ (althochdeutsch kolero: ‚Wut‘ von mittellateinisch cholera: ‚Zornausbruch‘, vgl. auch Choleriker) ist eine volkstümliche Eindeutschung und bezeichnet eine plötzlich aufbrechende oder stille, schwere psychologische Erregung, die sich aus einer Umgebung oder Situation ableiten lassen. Er äußert sich bei einzelnen Personen in Angst, Wut, Verzweiflung, Überaktivität sowie depressiven Zuständen.“^4^

Die jetzigen Quarantänemaßnahmen in Deutschland lassen sich zwar nicht mit einer Zwangsinhaftierung vergleichen. Dennoch kann man die in der psychologischen Forschung zu Extremsituationen gewonnenen Erkenntnisse nutzen, um den Umgang mit der aktuellen Situation zu erleichtern. Bereits durch die ungewohnt viele gemeinsame Zeit können Konflikte in der Partnerschaft oder im Familienleben entstehen. All dies kann sich in Streit bis hin zu Gewalthandlungen entladen. Folgende Maßnahmen sind präventiv hilfreich:

- Definieren Sie **klar abgegrenzte Stunden, die jede Person für sich allein verbringt**.
- Bieten Sie allen Familienmitgliedern **Rückzugsmöglichkeiten**. Machen Sie **allein einen Spaziergang** um den Häuserblock oder durch den Wald.
- **Sprechen Sie Ärger an, noch bevor die Situation eskaliert**. Kurzfristige Konflikte wird es bei jedem immer wieder mal geben – wichtig ist, dass diese gelöst werden.
- Halten Sie einen täglichen **Familienminikrisenstab oder eine Familienminikonferenz** ab: Wie geht’s allen Beteiligten? Wer braucht was? Welche Ideen und Wünsche haben die Einzelnen?
- **Seien Sie nachsichtiger als sonst, sich selbst und den anderen gegenüber!** Die derzeitige ungeplante soziale Isolierung mit „Zwangsferien“, die im Grunde gar keine Ferien sind, weil man nicht alles tun kann, was man im Urlaub gern tut, ist durchaus eine Herausforderung für alle Familien und Freundeskreise. Und wenn bei Ihnen mal „die Nerven durchgegangen sind“ und Sie einen Streit vom Zaun gebrochen haben oder aggressiv geworden sind, entschuldigen Sie sich, wenn sich die Situation wieder beruhigt hat (und nehmen Sie im umgekehrten Fall auch Entschuldigungen von anderen an) – auch dies kann sehr heilsam sein.

## Maßnahmen gegen Gewalt

Räumliche Enge, fehlende Rückzugsmöglichkeiten sowie der Mangel an Intimität können zu Aggression und Gewalt führen. Steuern Sie einer Eskalation der Situation aktiv und bewusst entgegen. Folgende Möglichkeiten haben Sie:

- **Erkennen und benennen Sie Gewalt.** Auch bei sich selbst. Gewalt hat viele Formen: Schlagen, Anschreien, Abwerten, längeres Ignorieren … Seien Sie sich selbst gegenüber ehrlich und reagieren Sie, wenn Sie merken, dass Sie selbst beginnen, vollkommen überfordert und in der Folge gewalttätig werden.
- **Telefonieren Sie zur eigenen Entlastung!** Telefonieren Sie mit einer befreundeten Person und sei es nur, um mal wieder mit jemand anderem zu sprechen. Wenn möglich, gehen Sie in ein anderes Zimmer. Atmen Sie tief durch.
- **Leben Sie Gewalt nicht aus!** Negative Emotionen, Anspannung und Aggressionen sind in Ausnahmesituationen normal. Es ist nicht schlimm, jemandem gegenüber aggressive Gefühle zu haben – gefährlich wird es erst, wenn man sie auslebt. Auch für Gewalt ausübende Menschen gibt es Beratungs- und Unterstützungsangebote dafür, solche Gewaltausübung zu unterbinden.
- **Wenn Gewalt passiert: Reden Sie!** Wenn Sie bemerken, dass andere Erwachsene zu Hause gewalttätig werden – gerade gegen Kinder oder Jugendliche –, reden Sie mit ihnen. Vielleicht sind Sie in dieser Situation die einzige Person, die jetzt den Schutz des Kindes herstellen kann. Lassen Sie sich dabei unterstützen: von der Telefonberatung eines Gewaltschutzzentrums, der Männerberatung, eines Kinderschutzzentrums, beim Krisendienst.
- **Holen Sie sich Hilfe, wenn Sie von Gewalt betroffen sind!** Hier ist wichtig, dass Sie nicht allein bleiben – denn Sie sind nicht allein, auch wenn es gerade in einer Isolationssituation so erscheinen mag. Holen Sie Hilfe: Bei Freunden, Beratungseinrichtungen, bei der Telefonberatung eines Gewaltschutz- oder Kinderschutzzentrums, im Fall von massiver Gewalt auch bei der Polizei oder der Kinder- und Jugendhilfe.
- **Und v.** **a.: Holen Sie sich rechtzeitig Hilfe!** Warten Sie nicht, bis es zu spät ist. Die vorangestellten Tipps gegen Langeweile, gegen Ängste und Sorgen und v. a. die Tipps gegen Konflikte helfen, mit den unangenehmen Gefühlen umzugehen, die in angespannten, oft beengten Situationen entstehen, bevor diese sich in Gewalt entladen.

*Diese gesammelten Hinweise weisen uns darauf hin, dass die derzeitige Krise nicht nur eine körperlich-medizinische (und in der Folge auch wirtschaftliche) ist. Auch die Psychologie, die Einstellung und das Verhalten aller, hat einen großen Einfluss darauf, wie wir als Gesellschaft diese zuvor nicht dagewesene Herausforderung bewältigen werden!*

Es sind nicht die Dinge selbst, die uns beunruhigen, sondern die Vorstellungen und Sichtweisen zu den Dingen^5^. Auch wenn politische Entscheidungen z. B. zur Einschränkung unserer Freiheiten kritisch beobachtet gehören und wir bei entsprechenden Fehlentwicklungen selbstverständlich die Stimme erheben sollen – jeder und jede kann selbst dafür Verantwortung übernehmen, dass die Situation nicht unerträglich wird. Zusammenhalt und Solidarität, Besonnenheit und eine trotz aller Belastungen optimistische Sicht auf die Dinge („positives ‚framing‘“) können massiv dazu beitragen, das Ausmaß der Pandemie und ihrer Folgen zu begrenzen.

**Wünschen wir uns allen dabei viel Erfolg und gutes Durchhaltevermögen!**

## Footnotes

1 RKI-Präsident Lothar Wieler in Podcast *Der Tag* (18.03.2020); https://www.deutschlandfunk.de/der-tag-mental-gesund-in-krisenzeiten.3415.de.html?dram:article_id=472763.

2 Hinweise zu entsprechenden klinischen Diagnosen siehe ICD (Kapitel V); https://www.dimdi.de/static/de/klassifikationen/icd/icd-10-gm/kode-suche/htmlgm2018/block-f10-f19.htm.

4 https://de.wikipedia.org/wiki/Lagerkoller.

5 https://de.wikipedia.org/wiki/Kognitive_Verhaltenstherapie.
